# Rapid assessment response (RAR) study: drug use and health risk - Pretoria, South Africa

**DOI:** 10.1186/1477-7517-8-14

**Published:** 2011-06-01

**Authors:** Monika ML dos Santos, Franz Trautmann, John-Peter Kools

**Affiliations:** 1 Strategic Information Department: Treatment Cluster: Foundation for Professional Development, PO Box 75324, Lynnwood Ridge, Pretoria, 0040, South Africa; 2 Head Unit: International Affairs, PO Box 725, NL - 3521 VS Utrecht, Trimbos Institute, The Netherlands; 3 International Liason, PO Box 725, NL - 3521 VS Utrecht, Trimbos Institute, The Netherlands

## Abstract

**Background:**

Within a ten year period South Africa has developed a substantial illicit drug market. Data on HIV risk among drug using populations clearly indicate high levels of HIV risk behaviour due to the sharing of injecting equipment and/or drug-related unprotected sex. While there is international evidence on and experience with adequate responses, limited responses addressing drug use and drug-use-related HIV and other health risks are witnessed in South Africa. This study aimed to explore the emerging problem of drug-related HIV transmission and to stimulate the development of adequate health services for the drug users, by linking international expertise and local research.

**Methods:**

A Rapid Assessment and Response (RAR) methodology was adopted for the study. For individual and focus group interviews a semi-structured questionnaire was utilised that addressed key issues. Interviews were conducted with a total of 84 key informant (KI) participants, 63 drug user KI participants (49 males, 14 females) and 21 KI service providers (8 male, 13 female).

**Results and Discussion:**

Adverse living conditions and poor education levels were cited as making access to treatment harder, especially for those living in disadvantaged areas. Heroin was found to be the substance most available and used in a problematic way within the Pretoria area. Participants were not fully aware of the concrete health risks involved in drug use, and the vague ideas held appear not to allow for concrete measures to protect themselves. Knowledge with regards to substance related HIV/AIDS transmission is not yet widespread, with some information sources disseminating incorrect or unspecific information.

**Conclusions:**

The implementation of pragmatic harm-reduction and other evidence-based public health care policies that are designed to reduce the harmful consequences associated with substance use and HIV/AIDS should be considered. HIV testing and treatment services also need to be made available in places accessed by drug users.

## Introduction

Recent data demonstrates that drug-related health problems (such as HIV infections) are increasing in South Africa. Within a ten year period South Africa has developed a substantial illicit drug market. There is evidence of increasing availability of illicit drugs (e.g. heroin, cocaine and methamphetamine) and growing drug-using populations from different social and ethnic backgrounds in different regions of the country [1,2]. Local research indicates ongoing spread of drug use, lowering age of starting drug users, increasing numbers of female users and spread of heroin use, especially in poorer black communities [1,2]. A study that was undertaken among three high risk and vulnerable populations (men having sex with men, sex workers and injecting drug users) in Cape Town, Durban and Pretoria was the first study in South Africa that elaborates on vulnerable groups and describes the fact that these populations are largely ignored by existing HIV responses. It highlights high risk behavior and the need for prioritizing interventions recognizing the role of drug use in HIV transmission and the need to address issues of access to services, stigma and discrimination [3-5]. Data on HIV risk among drug using populations clearly indicate high levels of HIV risk behaviour due to the sharing of injecting equipment and/or drug-related unprotected sex, as was found in the rapid assessment of drug use and sexual HIV risk patterns among vulnerable drug-using populations in Cape Town, Durban and Pretoria [4]. While there is international evidence on and experience with adequate responses, limited responses addressing drug use and drug-use-related HIV and other health risks are witnessed in South Africa. Government HIV intervention efforts have focused on the general population and two key high risk groups, namely youth and pregnant women. Limited attention has been given to preventing HIV among drug users [6]. A recent inventory on required needs and responses to address the drug-driven HIV risk underlines the necessity of developing adequate health services for drug using communities. This inventory, commissioned by the Dutch Embassy in South Africa in 2009, lists a need for effective health services to reduce drug related harm [7].

Injecting drug use is an increasing cause of HIV transmission, the number of countries in which injection of drugs has been reported has increased over the last decade. The high prevalence of HIV among many populations of injecting drug users represents a substantial global health challenge. Extrapolated estimates suggest that 15.9 million people might inject drugs worldwide. However, existing data are far from adequate, in both quality and quantity, particularity in view of the increasing importance of injecting drug use as a mode of HIV transmission in many regions such as South Africa [8]. Although injection drug use is low in South Africa in comparison with many other countries, with the increase over time in the use of substances such as heroin, the potential exists for this to change rapidly [9]. The rapid assessment undertaken with drug using commercial sex workers in Cape Town, Durban and Pretoria by Parry et al. in 2009 recognises the need for prioritising interventions recognising the role of drug abuse in HIV transmission, the issues of access to services, stigma and power relations [3]. Furthermore, a study by Dos Santos, Rataemane, Fourie and Trathen (2010) notes that limited strategic public health care policies that address substance use disorder syndromes complexities have been implemented within the South African context [10]. The study further emphasises the need for pragmatic and evidence-based public health care policies that are designed to reduce the harmful consequences associated with heroin use in particular, still needs to be implemented. According to Weich, Perkel, Van Zyl, Rataemane, and Naidoo (2008), medical practitioners in South Africa are increasingly confronted with requests to treat patients with heroin use disorders for example, but many do not posses the required skills to deal with these patients effectively [11]. The study by Dos Santos et al (2010) further discerns the need be make HIV testing and treatment services available in places accessed by vulnerable people as fear of stigma and discrimination often keep injecting users away from public health facilities [10]. According to Parry et al (2008) there is also a widespread lack of awareness about where to access HIV treatment and preventative services, and numerous barriers to accessing appropriate HIV and drug-intervention services such as long waits and appointments being cancelled without notice [4]. These authors further reiterate that multiple risk behaviours of vulnerable populations and lack of access to HIV prevention services could accelerate the diffusion of HIV.

The findings and recommendations from the assessment of the current drug/HIV situation in Pretoria, South Africa, are presented in this article. It forms part of the project *'Developing HIV prevention services among drug using populations and among prisoners in South Africa' *of the Trimbos Institute - the Netherlands Institute of Mental Health and Addiction, in cooperation with local South African partners. The project was implemented from September 2009 until October 2010 and was funded by the Dutch AIDS Fonds. The project of three assessments on the nature and extent of health problems among (injecting) drug users in Cape Town, Johannesburg and Pretoria. In Pretoria the assessment was undertaken by the Foundation for Professional Development (FPD)^2 ^with support from the Trimbos Institute. FPD is a South African Private Institution of Higher Education established in October 1997 by the South African Medical Association (SAMA) with support from the Trimbos Institute. Pretoria is the executive capital of South Africa with over two million inhabitants. The Pretoria drug scene can be described as emerging, with relatively large numbers (several hundred) of drug users, mainly black people, visiting and loitering in the inner city. Pretoria has a regional retail function for the large surrounding townships of Atteridgevile, Soshanguve, Mamelodi and the wider region.

The aim of this project was to respond to and address the rapidly emerging problem of drug-related HIV contraction and to stimulate the development of adequate health services for the drug users in South Africa, by linking international expertise and local research.

## Methodology

A rapid assessment response (RAR) methodology was adopted for the study, which included observation, reviewing existing information, mapping of service providers (SP), key informant (KI) interviews and focus groups (FGs). The assessment tool is based on the Rapid Assessment and Response Guide on Injecting Drug Use (IDU-RAR), the second author of this article has been actively involved in developing these international standards over the course of numerous years [12]. RAR methodology was initially developed by the Centre for Research on Drugs and Health Behaviour at the University of London for WHO and UNAIDS, and had been tested in various WHO projects in the field of drugs and addition - and is particularly suitable for investing problems within public health without resorting to 'unscientific' speculation, and which at the same time provides instruments and data for concrete intervention planning [13-17]. Since 1997 the approach has been tested extensively in developing and transitional countries in different regions around the world. This testing has been carried out by employing implementation strategies including training and consultancy, in order to support the implementation of assessments and subsequent intervention developments, using a draft version of the RAR Guide [18]. RAR's are thus used to collect relevant information for developing tailor-made health intervention and to assist in making decisions about appropriate interventions for health-related and social problems. This approach links the assessment of the nature and extent of a problem to the development of appropriate responses. An important characteristic of RAR is its ability to obtain a reliable picture in a short period of time by using multiple indicators and data sources. This data triangulation helps to obtain a reliable picture of the current situation in Pretoria. It also combines different methods to collect data, thus avoiding and correcting biases of a single source of information that might cover only part of the phenomenon investigated. It provides a more complete picture, including context information, which facilitates a better understanding of complex phenomena. The incorporation of views from varying stakeholders with differing backgrounds, both state and NGO, was adopted for the study [17].

The focus of RAR is on adequacy rather than scientific perfection. For adequate interventions in the field of health promotion the need to know the absolute number of people involved in certain risk behaviour is not necessary. It is sufficient to have cognisance that a substantial number of people are involved in this risk behaviour. Through cross-checking information from various data sources, RAR enables the establishment of reliable information about the occurrence and the nature of certain forms of risk behaviour. RAR is therefore used in cases where the focus is not on knowledge as such, but on knowledge which makes a quick response possible. Relevance to interventions and pragmatism are key features of RAR [17].

The following steps were included in the RAR: viewing existing information, access (to relevant stakeholders and target groups) and sampling (KI participants for interviews), semi-structured interviews, and FGs (to verify collected information and to agree on appropriate and feasible interventions).

To structure and organise the RAR a set of key questions was developed, comprising central questions for collecting information about both substance use and adequate responses. These form the basis and framework for all phases of information collection, i.e. for the development of the questionnaire in which these key questions are broken down into more detailed (sub) questions. The following set of key questions was adopted: 1) Who is using substances in a problematic way? 2) What substances are used? 3) How are substances used? 4) What health risks are involved in substance use? 5) What do substance users know about these health risks? 6) What do substance users do to avoid these health risks? 7) What interventions/services are available? 7) What interventions/services are realisable? Questionnaires were completed anonymously and all KI participants provided informed consent prior to the commencing the interview. Ethical approval for the study was obtained from a Medical Ethics Committee - METIGG Research Ethics Committee (The Netherlands) and the Foundation for Professional Development Research Ethics Committee (South Africa) in May 2010.

### Examining existing information

Consulting existing information within the South African context was the first step in the RAR process, much of this information has been included in the introduction of the article. Existing information included research articles and reports, reports prepared by health and drug services and information in the media. Reviewing the existing information assisted in the identification of possible gaps in the information, assisted in viewing information that can assist in monitoring changes over time and useful background information was gained from assessing the value or bias of findings.

### Access and sampling

The study commenced with the contacting of key informants (KIs) in the Pretoria area who were knowledgeable about substance use and related health and social problems (primarily service professionals who work with substance users). These KIs assisted with the identifying of further KI participants, both from the drug user population and from the service provider population. Mapping was implemented in order to identify potential points of entry and access. Purposive and snowball sampling was utilised in the study, defining beforehand relevant characteristics for the selection of both samples. For the selection of drug using participants the following characteristics were taken into consideration; gender, age, ethnic background and geographic location.

The following institutions were approached for the involvement of both users and service providers in the study: Vista Clinic (private psychiatric hospital), Denmar Clinic (private psychiatric hospital), Stabilis Treatment Centre (private rehabilitation centre), South African National Council on Alcoholism and Drug Dependence (SANCA) Pretoria: Castle Carey Clinic (private rehabilitation centre), Dr Fabian and Florence Rebeiro Treatment Centre (state rehabilitation centre), X-treme Freedom (Faith-based rehabilitation centre), Narcotics Anonymous, Tough*Love *(international support group programmes designed for parents in crisis in face of their teenager's behaviour), South African Police Service (SAPS), various private schools in the Pretoria and Foundation for Professional Development (academic organisation). The selection of these intuitions was decided on as they spanned the entire area of Pretoria, including Pretoria North, Central, Akasia and Centurion, furthermore, they represent all the major service providers for substance users in the Pretoria. The first author made contact with all the above-mentioned organizations and centers as she has extensive therapeutic and academic experience in working in some of the facilities, and networking with the various stakeholders.

The private psychiatric facilities in Pretoria and private schools that were approached to participate in the study declined as they regarded the key areas or research not to be in their scope of practice. The majority of KI user participants were recruited from the state rehabilitation centre and the faith-based rehabilitation centre. The KI user participants were thus in a process of attempting to remain abstinent from substances of abuse and were at the time of participating in the study undergoing residential treatment, this sample thus cannot be generalised to all substance using people in Pretoria as active drug users on the street, for example, were not sampled. KI users and service provider participants were also recruited from the private rehabilitation centres. Most KI user participants were thus interviewed in the facility that they were undergoing treatment in. Narcotics Anonymous and Tough*Love *also participated, as well as KI users not involved in any specific network. These participants were either interviewed at their homes, or at a NA meeting venue which was primarily at a Methodist church. SAPS in Sunnyside, Pretoria, was approached to participate in the study, and although they agreed to participate in the study, this never realised as the majority of police men and women were involved in the FIFA World Cup at the time of the implementation of the study. This can be regarded as one of the limitations of the study and could not have been predicted during the planning phase of the RAR. The security worker strikes at the various soccer stadiums across South Africa posed a serious security threat to the Soccer World Cup and the SAPS had to step in as an emergency measure in order to provide security at the stadiums (including the one in Pretoria) - this barrier could not have been foreseen. Fortunately, the Soccer World Cup did not impact on any other facets of the study. The study was implemented in Pretoria from May 2010 to July 2010.

### Semi-structured and structured interviews

For the individual interviews a semi-structured questionnaire was utlised that addressed issues covered by the key questions presented. Individual interviews were held with a total of 84 KI participants, 63 KI drug users (49 males, 14 females) and individual and FG consultations with 21 KI service provider participants from services and organisations (8 males, 13 females) (see Table 1 and Table 2 below). The information collected through the questionnaires served as background information for the FG with the selected stakeholders participants. Two research consultation psychology masters students and the first author were trained in the RAR methodology by Trimbos Institute, and conducted all the interviews and data analysis for the study. KI user participants were referred to treatment and other services as required. All KI participants remained anonymous for the purposes of the study and no names were documented on the questionnaires. A separate list with participants names and allocated codes was kept by the study leader and is being kept in a locked venue for a minimum period of three years.

**Table 1:** User Participants

Characteristic (users)	%		*n *	N *= *63
Gender				
Male	78		49	
Female	22		14	
Ethnicity				
White	65		41	
Black/	30		19	
Coloured	5		3	
Predominant substance - heroin	75		47	
Age (years)		(16-51)		

**Table 2:** Service Provider Participants

Characteristics (Service Providers)	%	*n *	N *= *21
**Gender**			
Male	38	8	
Female	62	13	
**Disciples represented**			
Psychology - 1	Social Work - 7	Lay counsellor - 4	
Tough*Love*/Parent - 4	Spiritual counsellor - 1	Nursing - 4	

### Focus groups

As a means of corroborating results in the concluding stage of the process, two different types of FG were initially planned, the first to verify collected information and to find explanations for diverging or contradictive information, and the second to reach consensus on appropriate and feasible interventions. As little diverging or contradictive information was found in the study outcomes, one mix of a FG type 1 and 2 was ultimately conducted. Due to venue constraints at FPD and the Royal Netherlands Embassy in Pretoria, the Centre for Disease Control and Prevention (CDC) Pretoria branch made a venue available for the FG meeting. KI service provider/stakeholders from different backgrounds were selected so as to encourage the imparting of their expertise on the various topics and proposed recommendations and actions. Bringing together a group of expert and target group representatives and discussing the outcomes of the RAR concerning adequate preventive responses to the problems founds, assisted in making plans more solid. It further assisted in obtaining the necessary commitments from relevant individuals to implement interventions successfully.

### Data analysis

Thematic content analysis was employed to analyse the interview information, such methods have been shown to be particularly valuable in the development of public health care interventions. Thematic coding was adopted in the analysis for the disaggregation of core themes, it is a multi-step process of during qualitative data analysis, that encompasses a process of relating codes (categories and concepts) to each other, via a combination of inductive and deductive thinking [19,20].

Responses were read, subjected to thematic content analysis by the first author, and discussed by all the authors in order to determine the usability of the material. Categories were established by removing the meaning units from the rest of the interview and applying phrases that would encompass several of these units at once in their totality. These categories were coded in order to identify the regularities. Categories that were clustered together became themes. The inductive categorisng of themes within the interviews increased the inter-rater reliability of the study. To authenticate interpretations, study conclusions were taken back to a sub-set of participants for enrichment and verification of interpretations, this input was obtained in the FG that was held after a draft report on the findings was compiled [19,20].

## 3. Results

### Specific characteristic impacting on substance use

Most KI user and service provider participants were of the opinion that ethnicity did not play a specific role in substance use; however, some held the view that blacks from lower income groups were more at risk to substance use. Over two-thirds of KI user participants and half of the KI service provider participants were also of the opinion that gender also does not play a specific or significant role in substance use. Notwithstanding, some of these participants felt that more males misuse substances.

Various KI user participants stated that coming out of an abusive home predisposes an individual to substance use, while a smaller minority of KI user and service provider participants were of the opinion that living conditions do not play a specific role in terms of substance misuse. Living with other users was also thought to predispose individuals to substance use. Informants were of the opinion that poorer communities are more susceptible to substance use.

The interlink between poverty and low education levels was thought by most informants to play a significant role in individuals developing as substance use problem. Various KI service provider participants were also of the opinion that poverty makes access to treatment harder. The KI user participants felt that vulnerability level and emotional/psychological characteristics could contribute to the development to a substance use problem. Approximately a quarter of KI user participants mentioned that African communities, especially men, were the most affected group, followed then by all the other population groups, the youth, young adults and the unemployed.

#### Substances used in a problematic way and availability

Over two-thirds of the KI user participants were of the opinion that heroin is used most in a problematic way, when mixed with cannabis (nyeope - smoked with cannabis, a South African township-culture term) this number increased further, with cannabis and cocaine following. The KI service provider participants also felt that heroin (alone and mixed with cannabis) was most used in a problematic way.

Cannabis, alcohol and cocaine/crack were also mentioned as substances used problematically, as these substances are all dependency producing and have various negative physiological and psychological side effects. Both groups of informants noted that heroin, cocaine, cannabis, crack cocaine and alcohol were the substances most easily available in the past 12 months in the Pretoria area. The opinion was held by just over a third of the KI user participants that all substances have become more easily available in the past 12 months in Pretoria, with cheaper forms of heroin mentioned in particular as being more available and cheaper forms by both groups of KI participants.

#### Substances preferred by specific groups, preferences and combinations of substances used

The KI user participants were of the opinion that African groups prefer cannabis, while crack cocaine is used by all groups. Heroin was thought to be mainly used by blacks and whites, while alcohol preferred by all groups. The KI service provider participants felt that heroin was mainly used by young adults, and that crack cocaine was primarily used by young adults of both gender.

The KI user participants further regarded heroin/cannabis and heroin/crack to be the substances most commonly combined, and that these combinations are sometimes used together with cannabis, alcohol and sedatives. Heroin and cannabis combined was also mentioned by the KI user participants, as well as the combination of cocaine/alcohol and the combination and crystal methamphetamine/other stimulants. The KI service provider participants mentioned heroin/cannabis combining as prominent, as well as heroin/cocaine and cannabis/Mandrax (sleeping tablet, contains methaqualone and diphrenhydramine) and cannabis/alcohol. The KI user participants cited club drugs (kat, ecstasy, cocaine, alcohol, GHB and LSD) as the most widely used combination of substances, followed by the crack cocaine/heroin combination, heroin/cannabis and alcohol/cannabis. The KI user participants felt that the combination of heroin/cannabis was used primarily by Africans, many of whom reside in the township area, and that the combination of heroin/crack is primarily used by white youth. KI service provider participants were generally of the opinion that the heroin/cannabis combination is used mainly by youth and adults.

#### Common routes of administration of substances

Both groups of KIs noted that heroin was most commonly smoked and injected (and less commonly snorted) in the Pretoria area, with cannabis and crack cocaine most commonly smoked. The KI user participants felt that blacks most commonly smoke heroin and whites are more likely to inject the drug. KI user participants also mentioned cannabis, with smoking the most common mode of ingestion for both Africans and whites. KI service provider participants cited cannabis as most commonly smoked, especially by those living in adverse conditions. Crack cocaine was also mentioned, with smoking as the most common mode of ingestion for both genders and amongst all races and ages.

#### Certain habits/patterns when using substances

KI user participants were of the opinion that in terms of heroin, whites most commonly engage in sexual risky behaviour and share needles, while blacks have a tendency to mix heroin with cannabis and smoke it. The KI user participants stated that cannabis is most commonly used by black males (sometimes mixed with Mandrax) and smoked in groups. Coloureds were also thought to most commonly smoke cannabis in groups. The KI service provider participants felt that heroin was most used by both genders, usually used alone and with many begging or stealing for money. Crime and sex work was also regarded to be pervasive in this population group, in order to feed users' habit. Crack cocaine was mentioned by the KI user participants, and the consensus was that it is most regularly used in groups.

#### The five most important risks of direct health damage due to substance use and knowledge of risks

Both KI participants felt that organ damage risks were the most direct health damage caused by substance use, as well as HIV/AIDS and STD transmission and mental disorders. Organ damage related primarily to liver, brain and kidney damage, while mental disorders referred mostly to mood disorders such as depression and psychotic disorders.

A third of the KI user participants mentioned that the majority of substance users were not concerned about their use, as many knew of organ/health problems and other direct risks of substance use - but were uncertain as to whether or not this could happen to them. Approximately a third of KI users also knew nothing or very little with regards to the direct health risks of substances. KI service provider participants felt that users generally knew about the direct risks of substances due to the available information and education, but felt that many do not care about the dangers due to the nature of their syndrome. Others said that users do not know about direct health damages, or have limited knowledge.

#### Knowledge on health risks for specific groups

Most KI user participants cited that regarding organ failure lack of knowledge remains a problem, while another proportion of KI user participants were of the opinion that organ failure was a myth that couldn't happened to them Approximately a third of the KI user participants were of the opinion that organ damage knowledge is obtained mainly from schools, the media, rehabilitation centres, other users, family and faith based organisations, and that the upper class had more access to the needed knowledge and services. It was further felt that knowledge with regards to HIV transmission is not yet widespread. KI service provider participants stated that those at most risk of HIV transmission were hyperactive young adults - but that they do obtain some degree of risk awareness while at school and from television. Those using heroin in particular were felt to know about health risks related to using heroin, but that due to the nature of their dependence, appeared not to care. Health damage knowledge was regarded to be scant, although some users were thought to obtain knowledge from faith based organisations, family, media, hospitals/clinics, and health care professionals.

#### What substance users do to protect themselves against health risks

KI user participants stated that organ damage can be prevented through taking multivitamins, referral to a medical practitioner, changing methods of using the substance and to stop using altogether. Participants further stated that HIV protection was insufficient, and that measures should be taken such as condom usage, using clean needles, stopping needle use altogether and to smoke instead of inject substances. Over two thirds of KI service provider participants generally felt that heroin users in particular do little if anything to stop using the substances or to protect themselves against related health risks, most attributed this factor due to the nature of heroin dependence and the ensuing ambivalence that many experience as a result of the psychological and physiological need for heroin versus the danger of using the drug.

#### What certain groups do to protect themselves

KI user participants stated that if whites experience any form of health damage or problems due to substance use they generally consult with medical practitioners, while blacks often consult with traditional healers and medical practitioners. The youth are also generally referred to health care practitioners should they experience organ damage or health problems. Generally informants felt that users know little regarding protective measures for organ damage, or they know of the damage but didn't care or where less cautious about their health during the course of their active substance dependence.

#### Interventions/services needed

Both groups of KIs generally felt that access to state sponsored treatment, including residential treatment, was needed, as well as more accessible private rehabilitation centers, media involvement, and drug awareness programmes in schools and jails. The need for therapeutic group work interventions, awareness and outreach campaigns with proper information, media information, police involvement and information sessions regarding the nature of substance dependence, relapses, grieving processes and crisis management was further identified.

KI user participants mentioned that some of the target group would accept all the intervention mentioned, however, a proportion of users were thought to maybe not accept all interventions due to denial of their problem, and due to the fact that they do not want to get caught and face potential criminal ramifications. Evangelical rehabilitation centers were also cited as not being accepted due to their extremist fundamentalist nature of their programmes as well as rehabilitation centers in general as the target group are often not prepared to go for treatment due to resistance and feeling forced to go. The high cost of attending rehabilitation centers was also cited as a factor that makes intervention not accepted.

A number of KI user participants felt that all services/interventions would be acceptable to politicians and policy makers. However, a number KI service provider participants mentioned that rehabilitations centers would not be acceptable to politicians and policy makers due to a lack of information and their unwillingness to provide funds. Others felt that not enough politicians and policy makers are trained in the field to make informed decisions. The KI user participants were of the opinion that all the services would be acceptable by the community; however, some mentioned that this is due to the fact that their backgrounds do not encourage critical thinking.

#### Organisations playing an important role in the available interventions/services

The KI user participants felt that NA/AA, rehabilitation centres and faith based organisations were playing an important role in making interventions available. Approximately half of the KI service provider participants cited the South African National Council on Alcoholism and Drug Dependence (SANCA) and government departments, such as social development, health, justice and correctional services as also playing an important role in the current availability of services and interventions for substance dependents. Conversely, KI user participants felt that local government, the private sector and faith based organisations should be getting involved in supporting and developing appropriate services for substance dependents, while the KI service provider participants stated that government departments and private organisations should be the primary role-players in developing appropriate services.

## Discussion

The rapid assessment findings indicate that both KI user and service provider participants felt that ethnicity and gender did not play a significant role in the development of a substance use problem. However, a significant number of KI user participants were of the opinion that black communities, especially men, were the most affected group within the Pretoria area. Adverse living conditions seemed to play a more prominent role in the development of such a problem, together with poor education levels. Poverty was also mentioned by the user participants as making access to treatment harder. Blacks, young adults and youth were cited as the most affected groups in terms of substance abuse, especially males. The availability of all substances was regarded to be on the increase in the Pretoria area over the course of the last twelve month, especially heroin (alone and mixed with cannabis).

Heroin was cited by both groups of KIs as the substance most used in a problematic way. Crack cocaine, alcohol and cannabis were other substances that were also mentioned as substances used in a problematic manner. The same substances were cited as those that have been most easily available in the Pretoria area in the last twelve months. No substantial corroboration could be obtained to discern clear differences in specific groups' preference for certain substances, however, heroin was thought to be used mainly by the black and white population group, while crack cocaine and cannabis is used by all population groups. The participants regarded heroin/cannabis and heroin/crack cocaine to be the substances most commonly combined, as well as cocaine/alcohol, various combinations of club drugs (kat, ecstasy, cocaine, alcohol, GHB and LSD) and alcohol/cannabis. The combination of heroin/cannabis was thought to be mainly used by young Africans in the township areas, and the combination of heroin/crack cocaine primarily by white male youth.

Heroin was cited to be most commonly injected and smoked, with blacks mostly smoking the substance and whites injecting it. Heroin was the only substance cited as being injected, however, injecting heroin remains less frequent compared to smoking heroin. Whites were also thought to engage in sexual risky behaviour and needle sharing when consuming heroin, finding of which are in agreement with the South African study of Morojele, Brook, and Kachienga (2006) and well as other international studies such as that of Semple, Patterson, and Grant (2004) [21,22]. Crime and sex work were associated more so with heroin use than any other substance, this might be due to the fact that heroin is more pervasively used than crack cocaine in the Pretoria area and/or that this may reflect the addictive nature of heroin use and the related high cost associated with it. Cannabis and crack cocaine were mentioned as being smoked with both substances being used across racial lines and used both genders, although cannabis smoking was more commonly associated with younger African males who often consume it in groups.

The direct risk of health/organ damage from substance use was overwhelming cited by all KI participants. Health and organ damage related to a range of problematic, including drain, liver and kidney damage, as well as skin lesions and abscesses. Affluence appears to play a prominent role in terms of accessibility to needed medical intervention. Overall HIV protection measures seems to be insufficient, and that more protective measures should be adopted, such as condom use, clean needle accessibility, and to stop using needles altogether. It appears as though both KI users and a number of KI service provider participants are also not fully aware of the real, concrete health risks involved in drug use, and the vague ideas that many participants hold does not allow for concrete measures to protect themselves (apart from ceasing drug use). This is underlined by some of the user participants citing multivitamin usage as an effective means of preventative intervention. This finding has important implications for responses on multiple levels: thorough information is urgently needed for users and professionals alike, such as information programmes and brochures (for users and service providers), training (for professionals), counseling, and peer support and education.

The mention of the development of mental disorders such as depression and psychosis highlights the need for integrated mental health services for those afflicted by the dual diagnosis of psychiatric disorders. Epidemiological studies have shown that between 30% and 60% of all substance dependents have a concurrent or co-morbid mental health diagnoses, including major depression, schizophrenia, bipolar disorder, anxiety disorders, PTSD and personality disorders [23-25]. A concurrent mental disorder can complicate substance use disorder treatment in a multitude of ways, for example, clinically depressed individuals have an exceptionally hard time resisting environmental cues to relapse. People with heroin dependence and mental illness co-morbidity, for example, are more likely to engage in behaviours that increase the risk of HIV/AIDS, and injecting heroin dependents with antisocial personality disorder more frequently share needles [26].

State sponsored interventions are also needed, especially residential care, as well as drug awareness campaigns in schools and correctional services, outreach programmes, legal enforcement and police intervention. It was also felt that the target group might not accept all interventions due to the denial of their problem, and due to the reality that they do not want to get caught by anyone. Evangelical religious rehabilitation centre interventions were also cited as not being accepted due to their fundamentalist and extremist strategies as well as rehabilitation centres in general as the target group may not prepared to go, some of these centers remain unregisterd in South Africa and various human rights violations have been reported [27]. The cost of residential treatment was regarded to be too high, and accessibility was regarded to be problematic. Similarly, in the study by Parry et al (2009), drug user interviewees felt that there was a shortage of drug rehabilitation centres, and suggested the opening of more drug treatment facilities in nearby areas as well as making more outreach programmes available [3]. The concern was further raised that rehabilitation centres would not be accepted by politicians and policy makers due to a lack of information and unwillingness to provide funding. The view was held that politicians and policy makers might not be trained extensively enough in the field to make informed decisions.

The following organisations were felt to be playing a significant role currently in the availability of services for substance dependents: NA/AA, rehabilitation centres, faith-based organisations, SANCA and government departments such as social development, health, justice and correctional services. There was consensus, however, that the various government and private sectors, as well as faith based organisations, could be playing a more proactive role in supporting and developing appropriate services for substance users.

A full-range of drug treatment and harm reduction measures were mentioned as being needed in order to assist users in protecting their health. Among KI service providers there was general consensus that harm reduction should be part of a full package of interventions *' with increasing numbers of kids on 'nyeope' we can't treat everyone, we also need harm reduction to keep them alive*', according to an service provider/pervious drug user KI informant from a faith-based treatment facility in Pretoria. The rapid assessment study by Parry et al. (2008) and various international studies also reported the existence of numerous barriers to the accessing and utilisation of risk reduction interventions [28,29].

The findings of this assessment are subject to limitations of the study design. Firstly, the sample size is relatively small and thus cannot be regarded as representative of the entire drug using or service provider population within the Pretoria area, furthermore, KI user participants were mainly recruited from intervention facilities/networks, thus users that did not fall in such networks (for example those on the street) were not sampled. The problems with the security worker strikes at the Soccer World Cup also prevented the SAPS from participating in the study. Although it is important bear in mind any potential sample representivity shortcomings, the aim of this rapid assessment was not to provide scientific perfection, but rather to focus on adequacy and to provide an explorative assessment of the current drug/HIV situation within the Pretoria area by utilizing multiple indicators and data sources, so that quicker recompenses can be developed and implemented within the Pretoria area. Systematic longitudinal research agendas making use of mixed designs with representational samples may go a long way in improving suggestion for intervention delivery.

## Conclusion and recommendations

The conclusions and recommendations of this article were formulated from the outcomes of both the RAR study and the FG group held after the completion of the initial study. Children, youth and young adults in particular who are not educated and who are economically disadvantaged are at a higher risk in terms of drug experimentation and drug use, education for children, youth and young adults can thus serve as a buffer again drug use. Education can also help shape proactive attitudes and behaviours amongst this high risk group. Special emphasis should be placed on prevention programmes by service providers targeting youth and young adults from abusive homes and youth that dwell in social surroundings in the Pretoria area where drug use is pervasive. Prevention programmes need to focus on HIV infection control and the development of knowledge and skills. Enhancing the efficacy of primary prevention and information campaigns aimed at different target groups; and enhancing the diversity, capacity and accessibility of prevention and treatment services, such as residential care and outreach programmes in Pretoria and nationwide, is further indicated.

Drug users in the Pretoria were not well aware of the real, concrete health risks involved in drug use. They held rather vague ideas which did not allow for concrete measures to protect themselves (except for quitting drug use). This of course has important implications for appropriate responses such as information programmes, leaflets, counseling, peer support and education, and medical/pharmacological education and intervention. Safer injecting messages need to be considered.

As highlighted in other studies local studies a thorough assessment to inform the care plan needs to be conducted [10,30]. Comorbidity concerns such as psychiatric illness need to be cogently taken into account, integrated in approach and addressed. Mental health and rehabilitation centres need to integrate modalities for intervention, as study outcomes indicate that some psychiatric facilities tend to see the aspects of substance dependence as not falling in their scope of practice, and vice versa relating to drug abuse rehabilitation centres. Physical, psychological, familial, social, cultural and spiritual factors need to be taken fully into account. Service providers should possess the right knowledge and skill to be of real help and needs to be applied effectively. As mirrored in the study by Dos Santos et al. (2010) the workforce needs to be expertly led, supervised and managed [10].

Taking into account the high prevalence of substance use within the African community, as indicated in other academic work and in the finding of this study, many African drug users consult with traditional healers, the collaboration between mental health practitioners and indigenous healers should also further explored, and specifically, what from of collaboration would be most appropriate [31].

Furthermore, advocacy is needed to convince politicians and policy makers of the need for rehabilitation programmes and other suitable responses. Adverse living conditions and poverty in the Pretoria area clearly needs to be addressed as this factor poses a high risk for substance misuse and also makes access to treatment more problematic.

HIV testing and treatment services in Pretoria need to be more widely advertised and made available in places accessed by vulnerable people. As corroborated in various studies, the fear of stigma and discrimination often keep (injecting) substance users away from public health facilities, and many drug users do now know where to access such treatment [10,3]. Active systems for auditing and monitoring processes and gaining client feedback should be encouraged, while the implementation of pragmatic and evidence-based public health care policies, such as needle exchange programmes, designed to reduce the harmful consequences associated with drug use and HIV/AIDS need to be considered for high risk areas in Pretoria.

## Competing interests

The authors declare that they have no competing interests.

## Authors' contributions

MMLDS drafted the original manuscript and assisted with the data collection and analysis together with the fieldworkers. FT and JPK advised and assisted in the interpretation of the data, technical quality of the paper and the development of policy recommendations based on the outcomes of the study. All authors, MMDS, FP and JPK, have read and approved the final manuscript.
